# Physiological consequences of nitrogen enrichment for corals in the Caribbean

**DOI:** 10.1098/rsos.250208

**Published:** 2025-06-25

**Authors:** Jonathan Jung, Ryanne Ardisana, Mark J. A. Vermeij, Erin L. Murphy

**Affiliations:** ^1^ Climate-Geochemistry Department, Max Planck Institute for Chemistry, Mainz 55128, Germany; ^2^ Department of Geology, University of Illinois Urbana-Champaign, Urbana, IL, USA; ^3^ Institute for Biodiversity and Ecosystem Dynamics, University of Amsterdam, Amsterdam, The Netherlands; ^4^ CARMABI, Willemstad, Netherlands Antilles; ^5^ Center for Biodiversity Outcomes, Arizona State University, Tempe, AZ, USA

**Keywords:** coral, nutrients, coral health, coastal pollution, conservation

## Abstract

Nutrient pollution has been a major contributor to coral decline throughout the Caribbean. Coral physiological responses to excess nutrients vary with nutrient forms (e.g. nitrate or ammonia), concentrations and nitrogen-to-phosphate (N : P) ratios. However, how these responses differ across nutrient contexts remains understudied. We show that *Orbicella annularis* photosymbiont densities respond differently to excess nitrogen in phosphorus-limited versus nitrogen-limited environments. Along Curaçao’s leeward reef, excess nitrogen significantly decreased (*p* < 0.05) photosymbiont density under phosphorus-limited conditions (N : P > 16) with low phosphorus (mean = 0.07 µM ± 0.06). In contrast, data from Barbados indicate a significant increase (*p* < 0.01) in photosymbiont density under nitrogen-limited conditions (N : P < 16). These findings highlight how nutrient contexts shape coral responses to nitrogen inputs, emphasizing the need to consider nutrient dynamics in coral conservation strategies.

## 1. Introduction

Reef building, scleractinian corals evolved to thrive in the nutrient-poor, oligotrophic waters of the tropics. Nutrient enrichment can reduce the health of corals either indirectly by promoting the growth of coral competitors, such as turf algae, or directly by altering coral physiology [1–4]. Both indirect and direct effects of nutrient enrichment can contribute to increased rates of coral bleaching, disease and mortality [5–8]; however, it can be difficult to disentangle the relative importance of indirect or direct effects. While the indirect effects on coral competitors have been scrutinized more extensively [8–10], direct physiological effects remain difficult to study because of context-dependent and often species-specific responses.

One of the most important physiological mechanisms impacted by nutrient enrichment is the symbiotic relationship between coral and single-celled photosynthetic dinoflagellates (‘photosymbionts’) [5,7]. Low nutrient concentrations regulate photosymbiont growth rates and maintain the stability of their mutualistic relationship with corals [11,12]. Nutrient enrichment can influence both, photosymbiont density and photosynthetic efficiency, thereby changing the energetic provisioning to the host [13,14] and potentially shifting the mutualistic relationship to a parasitic one [6,15]. However, the specific ways in which this symbiotic relationship might break down can depend on the ambient nutrient context the corals live in [16].

The production and remineralization of organic matter generally results in an oceanic nitrogen (N) to phosphorus (P) ratio of approximately 16 : 1, also known as the Redfield ratio (RR) [17]. Systems with N : P ratios greater than 16 : 1 are considered more P limited, while those with ratios less than 16 : 1 are considered N limited [18]. Deviances from the RR have been shown to directly influence photosymbiont densities [14,19–21]. Laboratory experiments conducted on a breadth of coral genera—*Acropora*, *Porites*, *Montipora*, *Stylophora*, *Pocillopora* and *Euphyllia*—have shown that N enrichment increases photosymbiont densities when N : P ratios remain below the RR, but that N enrichment decreases photosymbiont densities when N : P is greater than 16 [19,21–23]. However, N enrichment appears to only reduce photosymbiont densities when P concentrations were less than 0.18 µM, whereas densities can remain stable with P concentrations greater than 0.3 µM [24]. This shows the importance of considering not only N : P ratios [7,24,25] but also absolute P concentrations [21,24,26], when investigating the influence of nutrient limitation on coral health [16,27].

**Figure 1.**
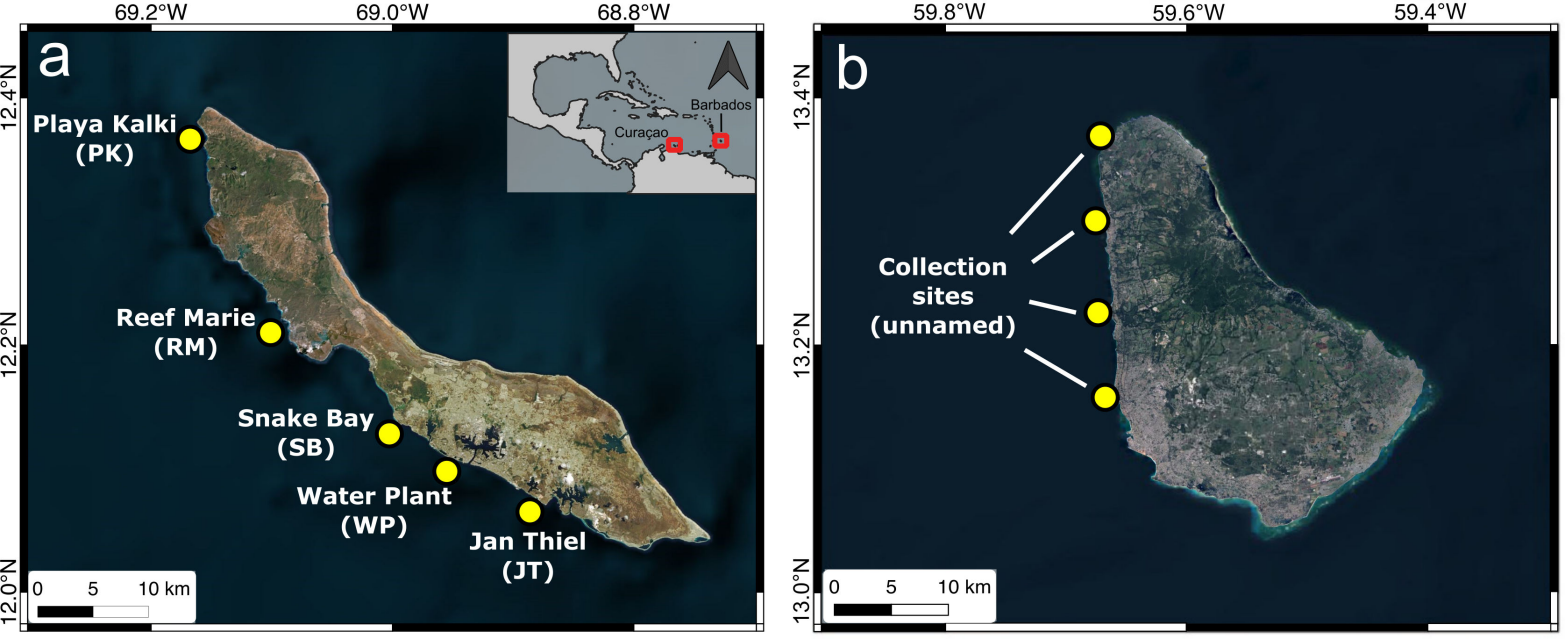
(a) Map depicting sampling sites along the leeward coast of Curaçao. The five study sites Jan Thiel (JT, 12.07°N, 68.88°W), Water Plant (WP, 12.11°N, 68.95°W), Snake Bay (SB, 12.14°N, 68.99°W), Reef Marie (RM, 12.22°N, 69.08°W) and Playa Kalki (PK, 12.37°N, 69.16°W) are indicated. (b) Comparative sites along the leeward coast of Barbados.

Despite several laboratory studies exploring the ways background N : P ratios and absolute P concentrations alter the effects of N enrichment on coral photosymbiosis, little research has been done to see if these responses also occur in natural environments. Here, we compare changes in coral photosymbiont densities in relation to gradients of excess N under two different underlying nutrient conditions. We used N : P ratios of ambient seawater to provide an environmental context in which the corals live. We documented photosymbiont densities of one of the last remaining widespread reef builders (*Orbicella annularis*) in the Caribbean, along a gradient of N enrichment on the western coast of Curaçao, a P-limited system, and compared our findings with previously reported photosymbiont density changes to N enrichment in *O. annularis* from Barbados, an N-limited system [14,28] (figure 1a,b). Our study confirms that coral photosymbiont densities decrease when subjected to N pulses while living in a P-limited system (Curaçao: avg. N : P > 41), while they increase when subjected to N pulses while living in an N-limited system (Barbados: avg. N : P < 14).

## 2. Methods

### 2.1. Study site and environmental conditions

The island of Curaçao (444 km^2^) has one of the most biodiverse marine ecosystems in the Caribbean [29]. Historically, Curaçao’s reefs have been subject to a variety of natural [30] and anthropogenic stressors [31–34]. Willemstad, the capital city, is home to approximately 80% of the nation’s population, and acts as a point source of many land-based pollutants [33]. In Curaçao, approximately 30% of wastewater is treated before discharge, with many rural households relying on cesspits, which can contaminate groundwater, across the island [35,36]. Finally, coastal communities in Curaçao are mainly built on porous limestone, which allows for water to easily seep through onto the reef and to alter nutrient ratios through predominant P adsorption on limestone [37,38].

The five study sites were Jan Thiel (JT, 12.07°N, −68.88°E), Water Plant (WP, 12.11°N, −68.95°E), Snake Bay (SB, 12.14°N, −68.99°E), Reef Marie (RM, 12.22°N, −69.08°E) and Playa Kalki (PK, 12.37°N, −69.16°E) (figure 1a, electronic supplementary material, figure S1). Per cent total N (%N) and δ^15^N of tissue samples of the abundant macroalgae *Dictyota* sp. were used as indicators of relative N enrichment and for N source attribution at each site, respectively. Algal tissue %N has been shown to reliably record time-integrated N loads in the coastal zone [19,39], while algal tissue δ^15^N can act as a proxy of human sewage pollution or fertilizer input [33,40]. Mean algal tissue %N and δ^15^N were based on the average of three measurements taken in 2015 by Sandin *et al.* [34] at each sampling site. Mean algal tissue %N was 1.81% ± 0.20 (±1 s.d.) at JT, 2.19% ± 0.41 at WP, 2.07% ± 0.28 at SB, 2.21% ± 0.26 at RM and 2.33% ± 0.23 at PK [34]. Mean algal tissue δ^15^N was 2.51 ± 0.31 at JT, 6.52 ± 0.53 at WP, 2.76 ± 0.12 at SB, 1.12 ± 0.39 at RM and 1.49 ± 0.12 at PK [34]. Algal %N was then calculated to average nitrate (NO_3_^−^) concentrations of the surrounding water column based on the empirical relationship,


$${NO_{3}}^{-}=10^{\left(Algal\;\%N\;-\;16.37\;/\;(6.15)\right)}*10,$$

given in Fong *et al.* [19].

### 2.2. Coral sample collection

In May 2014, three apparently healthy *O. annularis* colonies were sampled at each site for tissue and skeletal material at the reef flat between 6 and 8 m water depth. Two biopsies from three colonies at each study site were collected for analysis, using a McMaster-Carr steel core punch (2.5 cm diameter). Each sample included coral tissue and 4 cm of underlying skeleton. Samples were placed in a sterile 50 ml Falcon centrifuge tube flooded with seawater. Immediately, on return to the surface, tissue samples were stored in Formalin (9 : 1 DI-H_2_O : formaldehyde) at 4°C without light. Seawater depth and temperature were measured adjacent to each coral colony using an Oceanic Veo100 dive computer. Dive computers have been shown to have an accuracy of ±1.1°C [41], confirming colonies did not experience thermal stress at the time of sampling.

### 2.3. Coral tissue analysis

Tissue samples were imaged using two-photon confocal laser scanning microscopy (Zeiss TP-LSM710) following the methods developed by Sivaguru *et al.* [42,43]. Photosymbionts exhibit auto-fluorescence under UV excitation, emitting red light at 675 nm. This method does not require tissue fixation or alteration, such as thin sectioning. Intact coral biopsies were imaged at 20× magnification. Samples were optically thin sectioned, with images taken every 10 µm moving through the *z*-plane to ensure no photosymbiont cells were missed or double counted when three-dimensional tissue samples were reconstructed (electronic supplementary material, figure S2).

**Figure 2.**
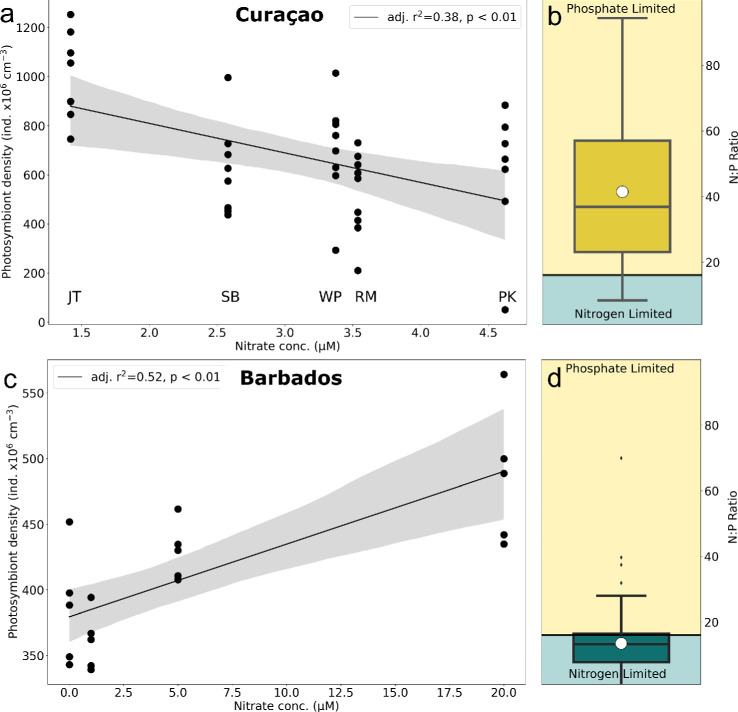
Photosymbiont densities (in indviduals × 10^6^ cm^−3^) of *O. annularis* corals from Curaçao and Barbados plotted against nitrate (NO_3_^−^) concentration (given in µM). (a) We observe a significant, negative correlation (adj. *r*^2^ = 0.38, *p* < 0.01) of photosymbiont density with increasing NO_3_^−^ concentrations at Curaçao. (b) In Curaçao, ambient seawater N : P ratios were above the RR of 16, indicating a P-limited system. (c) We observe a significant, positive correlation (adj. *r*^2^ = 0.52, *p* < 0.01) between previously measured photosymbiont density and increasing NO_3_^−^ concentrations at Barbados. (d) In Barbados, ambient seawater N : P ratios were below the RR at the time of analysis, indicative of an N-limited system [14,28].

IMARIS was used to recreate the three-dimensional image of each coral polyp to quantify average tissue abundance per colony [42]. Photosymbiont cell abundance was measured from three polyps on three colonies from tissue overlying secondary septa at five locations around the leeward side of Curaçao (a total of nine samples per location) (electronic supplementary material, figure S3). Tissue overlying secondary septa were used because they exhibited less damage associated with sample collection than primary septa. To measure cell abundance over each septum, the three-dimensional volume of the entire polyp was recreated, and the reconstruction was cropped to isolate the tissue over an individual secondary septum. Relative cell abundance was then parametrized as the volume of photosymbionts over the total volume of the secondary septa. Relative cell abundance for each colony is given and is presented as a per cent of total tissue volume. Finally, photosymbiont density was calculated using the photosymbiont fraction divided by average volume per photosymbiont (given in individuals × 10^6^ cm^-3^) (figure 2a,c). Chromatophore relative cell abundance was also assessed following the same methods to identify if there was a relationship between photosymbiont and chromatophore abundance (see electronic supplementary material for methods, results and interpretation of chromatophore tissue abundance).

For Barbados, previously reported photosymbiont densities in *O. annularis* were taken from [14]. Values are given in individuals × 10^6^ cm^-2^ and were transformed to cm^-3^ by using the thickness (0.1 mm) of the haemocytometer field used in this particular study.

### 2.4. Equatorial Atlantic and Caribbean N : P ratios

To constrain regional nutrient limitation, open ocean water samples were collected between December 2022 and March 2023 with a Rosette water sampler equipped with five 5 l Niskin bottles. The sampling was conducted along an east–west transect across the Atlantic at 13°N and across the Caribbean Sea at 11°N during a cruise aboard the research sail yacht *Eugen Seibold* (https://www.mpic.de/4224334/sy-eugen-seibold) [44]. The transect was designed to detect a gradient in ambient N : P ratios of subsurface seawater and is close to Barbados (13°10′N) and Curaçao (12°10′N).

All newly collected water samples were frozen upon collection and kept frozen at −21°C until analysis. For open ocean water samples, NO_3_^−^, nitrite (NO_2_^−^) and phosphate (PO_4_^3−^) analyses were conducted at the Max Planck Institute for Chemistry. NO_3_^−^ + NO_2_^−^ concentrations were first determined according to Braman & Hendrix [45] on a Teledyne API T200 NO_x_ analyser with a detection limit of 0.01 µM and a precision (±1 s.d., *n* = 55) of 0.55%. PO_4_^3−^ concentrations were determined on a continuous flow autoanalyser (QuAAtro, Seal Analytics) with a detection limit of 0.01 µM and a precision of 0.50% (±1 s.d., *n* = 55). For all analyses, certified reference materials (Kanso, Japan) were run alongside samples to determine recovery rates and calculate the daily limit of detection, defined as three times the standard deviation of the lowest standard.

Newly collected water samples were then compared with all available Atlantic and Caribbean nutrient data at 200 m compiled in the Glodapv2.2022 database. These data were based on cruises that have reliably measured NO_3_^−^ and PO_4_^3−^ that can serve to constrain regional water N : P context. Waters at 200 m were chosen as they record stable N : P ratios of the subsurface and are less likely to be influenced by changes in pycnocline depth [46].

### 2.5. Local N : P ratios

To understand local N : P ratios around Curaçao and their effect on coral physiology, coastal water samples were taken with a Niskin bottle from different locations in November 2015 at depths ranging from 10 to 67 m to compare coastal nutrients within the regional N : P context. Water was filtered through a 0.2 µM track-edged filter and 20 ml sample bottles were rinsed three times with filtered seawater before being stored at −20°C. On the day of analysis, samples were defrosted and analysed for nitrate (NO_3_^−^), nitrite (NO_2_^−^), ammonia (NH_4_^+^) and phosphate (PO_4_^3−^). Using these values, N : P ratios were calculated and an average N : P was taken for the island around the time of coral tissue sampling from 2013 to 2015 (figure 2b).

For Barbados, N : P ratios were pulled from the Glodapv.2.2022 database for water samples greater than 100 m. An average N : P ratio is given for the time range between 1981 and 1983, when the corals were sampled [14,28] (figure 2d).

### 2.6. Data analyses and representations

Statistical analyses were conducted in R (2022.02.2 + 485 ‘Prairie Trillium’ Release). A Bartlett’s test was used to confirm homogeneity of variances for all samples. ANOVA tests were used to assess differences between photosymbiont tissue abundances for different colonies at the same site (intra-site variation) and differences between sites (inter-site variation). Where significant results from the ANOVA test were found, a Tukey HSD test was then used to identify differences between sites for average photosymbiont tissue abundance. The relationship between nutrient concentration at each site and coral physiological responses was assessed using a linear regression model. *p*-values considered for significant differences were ess than 0.05. Average values are given with ±1 s.d. Figures were produced in Python3 on a Jupyter Notebook^®^ (v. 5.7.4). Data were imported using the Pandas library and plotted with Seaborn or Matplotlib libraries.

## 3. Results

### 3.1. Photosymbiont abundance

Average photosymbiont abundance varied between sample sites in Curaçao (ANOVA; *p* = 0.021) but did not vary significantly between colonies from the same sites in Curaçao (ANOVA, *p* > 0.05). Jan Thiel had the highest photosymbiont density (22.7% ± 6.47, Tukey HSD; *p* < 0.05) compared with all other sites—Snake Bay (16.64% ± 5.22), Reef Marie (16.74% ± 4.23) and Playa Kalki (15.22% ± 4.03)—which had similar relative abundances (*p* > 0.05) (figure 2a). Photosymbiont densities for each colony had a significant negative correlation with calculated NO_3_^−^ concentrations from each location (figure 2a, adj. *r*^2^ = 0.38, *p* = 0.0017), but showed no relationship with mean algal tissue δ^15^N (*p* > 0.05). Chromatophore tissue cell abundance showed no relationship with %N or δ^15^N (*p* > 0.05*,* see electronic supplementary material for results and discussion of chromatophore data).

For Barbados, photosymbiont densities were significantly positively correlated with increasing NO_3_^−^ concentrations (figure 2*c*, adj. r^2^ = 0.52, *p* = 0.00024).

### 3.2. Equatorial Atlantic and Caribbean N : P ratios

**Figure 3.**
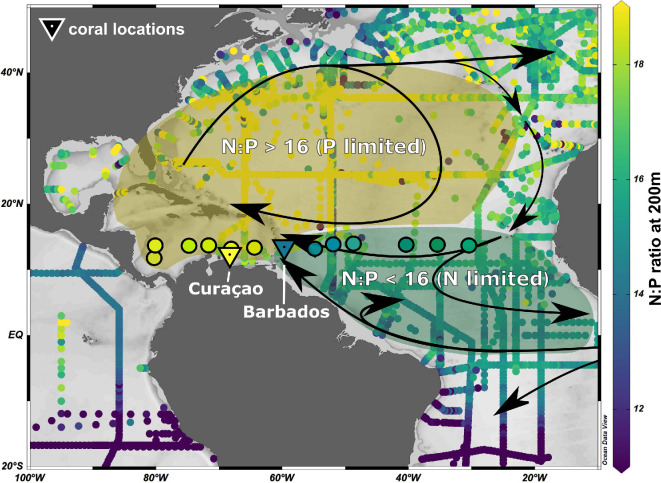
Atlantic and Caribbean N : P ratios from 200 m depth as calculated from nutrient measurements compiled in the Glodapv.2.2022 dataset. N : P ratios of water samples taken upon the research sail yacht *Eugen Seibold* are indicated as large circles. Coastal N : P data close to the respective *O. annularis* coral locations are given as triangles and plotted on the same colour range. All open ocean data are plotted as dot and each dot is based on cruises that have reliably measured NO_3_^−^ and PO_4_^3−^ in the region. Shaded areas are given and indicate an overall P-limited region in yellow versus an N-limited region in green.

Average open ocean N : P ratios at 200 m water depth for water samples taken upon the research sail yacht *Eugen Seibold* varied significantly between the equatorial North Atlantic and the Caribbean Sea (electronic supplementary material, figure S5). The equatorial North Atlantic showed lower average N : P values at 14.29 ± 5.93 compared with the Caribbean with average N : P values at 24.12 ± 4.17. Overall, N : P values align well with measurements compiled in the Glodapv2.2022 and show two distinct regions, whereby the Wider Caribbean Region shows N : P ratios greater than 16 which is indicative of a P-limited system and the equatorial North Atlantic shows N : P ratios less than 16 that are indicative of an N-limited region (figure 3).

### 3.3. Local N : P ratios

During the time of coral sampling, sea surface temperature (SST) was below the bleaching threshold (ess than 29°C) across all sites in Curaçao, allowing us to isolate the influence of excess N on the physiology of apparently healthy coral individuals. N : P ratios along the entire coast ranged from 8.34 to 94.42 between 2013 and 2015, with an average ratio of 41.46. Average total N (NO_3_^−^ + NO_2_^−^ + NH_4_^+^) along the entire reef tract was 3.21 µM ± 2.91, whereas average P (PO_4_^3−^) showed very low values of 0.07 µM ± 0.06 (figure 2b).

At the time of analysis in Barbados, average SST was lower at 27.45 ± 0.76°C. N : P ratios around Barbados ranged from 0.35 to 69.94 between 1981 and 1982, with an average of 13.50 ± 9.32 (figure 2d).

## 4. Discussion

Most scleractinian corals thrive in oligotrophic surface waters and can react sensitively to nutrient enrichment [25,47]. Nutrient pollution has been identified as one of the most important local contributors to water quality impairment in the Wider Caribbean region [48–52], and nitrogen enrichment plays a crucial role in shaping the health and resilience of reef communities across spatial and temporal scales [49,52–54]. Changes in water quality can alter host–symbiont dynamics and make corals more susceptible to bleaching and disease under predicted sea surface temperature rise [5,6,24,25]. In fact, reducing land-based stressors has been shown to result in a threefold to sixfold greater probability of sustaining coral cover after bleaching events [55]. However, previous work has stressed that nutrient addition has nuanced effects on coral physiology that are shaped by the underlying context of nutrient ratios and absolute nutrient concentrations [7,13,16].

Curaçao and Barbados offer an interesting comparison to study the influence of excess nutrients on the physiology of reef-building corals such as *O. annularis*. Although relatively close together, the islands occupy different oceanic basins with opposing regional N : P ratios (figure 3). As a result, comparing the influence of excess N in these two systems provides a better understanding of how natural differences in nutrient ratios can shape the response of corals to excess N.

During the time of our sampling, the reef tract in Curaçao was characterized by high N : P ratios and low absolute P concentrations that indicate P-limited conditions. Coral photosymbiont density is negatively correlated with increasing NO_3_^−^ concentrations along the western coast of Curaçao, while it was positively correlated with increasing NO_3_^−^ concentrations in Barbados (figure 2). These findings support several laboratory experiments in which the introduction of N increases photosymbiont density in N-limited systems [21], whereas photosymbiont densities decrease with N enrichment under P-limitation (N : P > 16) [6,7,13,24]. Therefore, our research provides *in situ* evidence that the same coral species reacts differently to N enrichment based on ambient N : P conditions (figures 2 and 3).

In N-limited environments, corals are capable of limiting photosymbiont densities by restricting ammonia (NH^4+^) recycling to their symbionts [56,57]. With N enrichment, photosymbionts are no longer N restricted and can multiply more rapidly, leading to increased symbiont densities and increased photosynthetic output within the coral host [6,14]. Although this can be beneficial to corals under normal conditions, under high temperature and light conditions more photosymbionts and resulting excess photosynthetic output can lead to an overproduction of reactive oxygen species (ROS) within the coral cells. The ROS generated by overactive photosynthesis can damage coral cell membranes, proteins and DNA, overwhelming the coral’s antioxidant defences [58,59]. To cope with the stress, corals can expel the photosymbionts to prevent further ROS damage and bleach more easily [7,21,60].

On the other hand, when P is limiting, symbionts lack sufficient P to maintain high concentrations of rRNA for cell division and growth rates [61,62]. As a result, photosymbionts use energy to prioritize the maintenance of photosynthetic activity and minimize cell proliferation [63]. With further N enrichment, P limitation can be exacerbated into P starvation and photosynthetic efficiency of remaining photosymbionts will eventually decrease [13,24]. Prolonged periods of low photosymbiont density can lead to energy depletion of the coral host and increased vulnerability to disease and mortality [26]. However, photosymbiont densities can be maintained under high N : P ratios (greater than or equal to 22 : 1) when absolute P concentrations are greater than 0.3 µM [24]. Therefore, excess N only leads to a decrease in photosymbiont density when P concentrations are very low (categorized as P < 0.18 µM) [24].

The high N : P ratios and low absolute P concentrations observed in Curaçao at the time of sampling explain the inverse relationship observed between additional N and a reduction in photosymbiont densities. While some research suggests photosymbiont density is always reduced in low P conditions [13,24], other research shows that photosymbionts are less sensitive to low P concentrations under more balanced N : P ratios (LNLP conditions) [63]. Photosynthetic efficiency is also less affected under balanced nutrient ratios [13,24], which suggests that the influence of nutrient enrichment on coral physiology changes in the context of natural or anthropogenic nutrient concentrations and N : P ratio variability. Combined, lower photosymbiont density and subsequent lower photosynthetic efficiency render the coral animal more susceptible to additional stressors if low P conditions are sustained.

However, our results reveal an asymmetric response of *O. annularis* photosymbiont densities to excess N across the two different nutrient regimes, with a greater magnitude of decline under P-limited conditions compared with the increase observed under N limitation (figure 2a,c). This suggests that N enrichment is particularly detrimental when P availability is low, probably due to an even more disrupted nutrient balance within the coral-algal symbiosis. The pronounced reduction in symbiont densities under high N : P conditions underscore the heightened vulnerability of corals in P-limited environments, a common condition on many Caribbean reefs.

The lack of relationship between N and δ^15^N of the same algae tissue samples suggests that the δ^15^N is decoupled from the N source along the reef tract. High δ^15^N values are found in samples collected at Water Plant close to Willemstad but are low in all other locations (electronic supplementary material, figure S6) [34]. Relative enrichments of δ^15^N have previously been associated with increased sewage input [33,49,64,65], increased denitrification [66,67] or with partial nutrient consumption [68]. The decoupling of %N and δ^15^N in the tissue of *Dictyota* spp. suggests that the δ^15^N signal is derived from processes related to Willemstad but that the bay is not the primary source of excess N. Instead, more diffuse sources, such as groundwater seepage, could influence the leeward reef tract of Curaçao and introduce significant amounts of N [37]. This is especially true for coastal areas with large limestone frameworks that adsorb P more readily [38,69,70], which leads to low absolute P concentrations, like those observed in this study.

Taken together, our results suggest that nutrient enrichment is having distinct physiological effects on apparently healthy *O. annularis* along the leeward coast of Curaçao, while photosymbiont density decreased along the Curaçao reef tract in response to N enrichment it showed opposite trends in response to N enrichment in Barbados (figure 2). The opposing photosymbiont density trends are probably the result of ambient N : P conditions and the absolute P concentrations of waters in which the corals live (figures 2 and 3).

### 4.1. Implications for local management

Our findings have implications for coral reef management throughout the Caribbean. The Western North Atlantic and Caribbean Sea are generally P limited [71–74] and anthropogenic activities close to shore, especially on large limestone frameworks, increase N disproportionally, thereby amplifying P-limitation [38,75]. Our results indicate that exposure to high N : P ratios coupled with low absolute P concentrations reduce relative photosymbiont densities in coral tissue, which has been shown to make corals more susceptible to bleaching [2,6,7,55] and disease [5]. As such, nutrient conditions can exacerbate the significant threat climate change is posing to Caribbean corals [7,76] and should be considered in coral conservation efforts.

## Data Availability

All data are made available on Dryad [77]. Supplementary material is available online [78].
